# YOLOv8-C2f-Faster-EMA: An Improved Underwater Trash Detection Model Based on YOLOv8

**DOI:** 10.3390/s24082483

**Published:** 2024-04-12

**Authors:** Jin Zhu, Tao Hu, Linhan Zheng, Nan Zhou, Huilin Ge, Zhichao Hong

**Affiliations:** 1 Ocean College, Jiangsu University of Science and Technology, Zhenjiang 212003, China; oscar@just.edu.cn (J.Z.); 221112202109@stu.just.edu.cn (L.Z.); zhounan@just.edu.cn (N.Z.); ghl1989@just.edu.cn (H.G.); 2 School of Naval Architecture and Ocean Engineering, Jiangsu University of Science and Technology, Zhenjiang 212003, China; 3 Jiangsu Marine Technology Innovation Center, Nantong 226007, China

**Keywords:** water contamination, underwater target detection, YOLOv8, remote sensing

## Abstract

Anthropogenic waste deposition in aquatic environments precipitates a decline in water quality, engendering pollution that adversely impacts human health, ecological integrity, and economic endeavors. The evolution of underwater robotic technologies heralds a new era in the timely identification and extraction of submerged litter, offering a proactive measure against the scourge of water pollution. This study introduces a refined YOLOv8-based algorithm tailored for the enhanced detection of small-scale underwater debris, aiming to mitigate the prevalent challenges of high miss and false detection rates in aquatic settings. The research presents the YOLOv8-C2f-Faster-EMA algorithm, which optimizes the backbone, neck layer, and C2f module for underwater characteristics and incorporates an effective attention mechanism. This algorithm improves the accuracy of underwater litter detection while simplifying the computational model. Empirical evidence underscores the superiority of this method over the conventional YOLOv8n framework, manifesting in a significant uplift in detection performance. Notably, the proposed method realized a 6.7% increase in precision (P), a 4.1% surge in recall (R), and a 5% enhancement in mean average precision (mAP). Transcending its foundational utility in marine conservation, this methodology harbors potential for subsequent integration into remote sensing ventures. Such an adaptation could substantially enhance the precision of detection models, particularly in the realm of localized surveillance, thereby broadening the scope of its applicability and impact.

## 1. Introduction

In the contemporary era, the management of waste has escalated to a critical environmental challenge, underscored by the burgeoning accumulation of refuse within the world’s aquatic ecosystems. Annually, an estimated 1.15 to 2.41 million tons of plastic debris are conveyed into the oceans via riverine systems, underscoring a pervasive conduit of pollution [1]. This deluge of plastic waste, fragmenting into minuscule particulates known as micro plastics, poses a latent threat to the biosphere [2]. These diminutive fragments harbor the potential to permeate through trophic levels, being ingested or inhaled by a diverse array of species, including humans, thereby insidiously infiltrating the food web. The ramifications of this phenomenon extend beyond the immediate ecological disturbances, catalyzing broader spectrums of water pollution and engendering a cascade of environmental and health-related adversities [3].

The advent of graphics processing unit (GPU) technology has expedited the progress of neural networks [4], with deep learning methodologies stemming from artificial neural networks [5]. In the field of deep learning target detection, there are two main categories: two-stage and single-stage [6]. The former includes R-CNN (Regions with Convolutional Neural Network Features) [7], Fast R-CNN [8], and Faster R-CNN [9], while You Only Look Once (YOLO) [10] and SSD (Single Multi-Box Detector) [11] are the most commonly used single-stage models. These approaches have been successful in the detection of targets on the road surface in conventional domains. In recent years, there has been an increase in the use of algorithms in underwater environments. However, progress in detecting underwater objects has been hampered by the inadequate availability of underwater datasets.

Underwater target recognition, as a burgeoning specialty divergent from traditional surface detection approaches, encapsulates the identification of diverse marine species and objects with notable challenges posed by the aquatic medium’s optical properties. The evolution of this field has been marked by significant advancements through deep learning and tailored network designs, as detailed below:

(1) Early Innovations:

SWIPE Net: Chen et al. [12] employed this novel network on the URPC2017 dataset featuring limited samples of sea cucumbers, urchins, and starfish, attaining a mAP of 46.3%.

Enhanced SSD and CNNs: By 2018, with the expansion of target classes in URPC2018, an improved mAP of 64.5% was recorded, and novel architectures like enhanced SSD by Jiang et al. [13] and deep CNNs by Han et al. [14] pushed mAPs to 66.9% and, notably, 91.2%, respectively.

(2) Integration with Existing Frameworks:

Faster R-CNN and YOLO Networks: Lin et al. [15] and Liu et al. [16] enhanced these renowned frameworks, integrating strategies like RoIMix and water quality assessment systems, with mAPs reaching 74.92% and 63.83% on subsequent datasets.

SA-FPN on PASCAL VOC: Xu et al. [17] took the SA-FPN to the esteemed PASCAL VOC dataset, achieving a high mAP of 76.27%, indicating the method’s cross-environment versatility.

(3) YOLO Series Innovations:

Continuous YOLO Improvements: From YOLOv3 enhancements to the underwater-specific YOLOv4-uw, these modifications led to mAP improvements, with YOLOv5 iterations by Wang et al. [18] and the YOLOv5s-CA by Wen et al. [19] achieving mAPs of 69.3% and 80.9%.

CME-YOLOv5 for Species Identification: Li et al. [20] introduced this model in 2022, which stood out for identifying distinctive fish species with a high mAP of 92.3%.

(4) Recent Multi-Attention and Coordination Approaches:

APAN and TC-Network Models: In the latest trends, Yu et al. [21] introduced APAN, while Liu et al. [22] proposed the TC-Network model, tackling real-world challenges with mAPs of 79.6% and 83.1%.

The trajectory of underwater target recognition showcases a shift from reliance on general deep learning models to more specialized networks attuned to the unique underwater environment. The trend reveals a keen focus on enhancing feature extraction, addressing light distortion, and balancing the clarity and color fidelity specific to underwater imaging. The aforementioned milestones underscore the progression toward more refined and precise recognition capabilities in marine settings.

The current predominant research challenge revolves around the issue of detecting small targets. Small targets are defined as those with dimensions less than 32 * 32 pixels, as categorized by the MS COCO 2014 [23] shared dataset. This challenge has been the focus of much attention, particularly since the advances of Lim et al. (2021), which introduced a contextual attention mechanism as a solution to the problem [24]. By 2022, Cheng et al. report that the WHO has adopted a strict area criterion: cases with an area of less than 1024 pixels are defined as small items [25]. Previous studies have shown that underwater lighting has a significant impact on the quality of underwater images [26]. However, in the context of underwater targets, due to occlusion and distance issues, they tend to be small and are therefore classified as small target objects. In order to facilitate the subsequent detection process of the net, it is essential to extract the limited number of features from such small targets during the feature extraction phase [11].

Substantial advancements have been realized in the sphere of Remotely Operated Vehicle (ROV) technology, encompassing a broad spectrum of applications and developmental strides. Yet, the domain of underwater litter detection remains relatively underexplored, presenting a notable gap in research. The task of precisely identifying diminutive submerged objects, minimizing false positives, and circumventing missed detections presents a formidable challenge. In this context, this study introduces a refined algorithm, YOLOv8-C2f-Faster-EMA, building upon the foundational YOLOv8 detection framework, tailored specifically for the identification of underwater trash. To address the dual concerns of false positives and negatives, this paper proposes three pivotal enhancements: the augmentation of the C2f module, the integration of multi-scale calibrated detection, and the fortification of the backbone network. The efficacy of the network was evaluated through the metric of mAP, utilizing the TRASH-ICRA19 dataset for training purposes to ascertain the algorithm’s performance. The empirical outcomes underscore a marked enhancement in network efficiency relative to comparator frameworks, evidenced by a 5% increment in average precision (AP) and a notable 6.7% uplift in ROV detection precision, heralding significant implications for the field of underwater environmental monitoring.

The YOLOv8 network has achieved a marked advancement in the detection of underwater trash, demonstrating significantly enhanced efficiency and precision. This breakthrough is poised to revolutionize the ability to pinpoint and discern diminutive objects within authentic subaqueous settings, and holds considerable promise for applications in remote sensing orientations. Such an evolution in detection technology underscores a pivotal stride forward in environmental monitoring and conservation, offering new vistas in our understanding and stewardship of aquatic ecosystems.

In this manuscript, we delineate three pivotal advancements within the domain:

(1) The introduction of the C2f-Faster-EMA module represents a paradigm shift aimed at augmenting the model’s perceptual discernment through the integration of multi-scale feature fusion and attention mechanisms. This enhancement is geared towards elevating the precision and robustness of target detection. Crucially, our investigation delves into the impact of the module’s spatial positioning within the network architecture, revealing that the locational variance of the same module can significantly influence detection outcomes. This insight lays the groundwork for future exploratory avenues.

(2) The amalgamation of FasterNet and Efficient Multiscale Attention (EMA) modules within the underwater trash detection schema marks a significant leap forward, evidenced by a notable 5% increase in AP. This achievement not only surpasses traditional methodologies but also sets a new benchmark in the field.

(3) Through a comprehensive suite of rigorous benchmarking experiments and evaluations, the superior performance of our innovative network is unequivocally demonstrated. The findings corroborate the method’s efficacy, showcasing an exceptional blend of efficiency and unparalleled performance vis à vis existing paradigms.

## 2. YOLOv8 Network Architecture

The YOLO network [10] is a popular real-time object detection system, first introduced by Joseph Redmon and colleagues in 2016. The design of this network enables it to recognize objects within an image in a single scan, a stark contrast to previous methods that required multiple scans. The advent of YOLO marked a significant breakthrough in the field of computer vision, renowned for its rapid processing and high efficiency. In January 2023, Ultralytics released YOLOv8, further expanding the YOLO series. YOLOv8 offers multiple versions to support a variety of visual tasks and utilizes a backbone network similar to that of YOLOv5. It features the newly introduced C2f module, which enhances the integration of features with contextual information, thereby improving detection accuracy.

**Figure 1:**
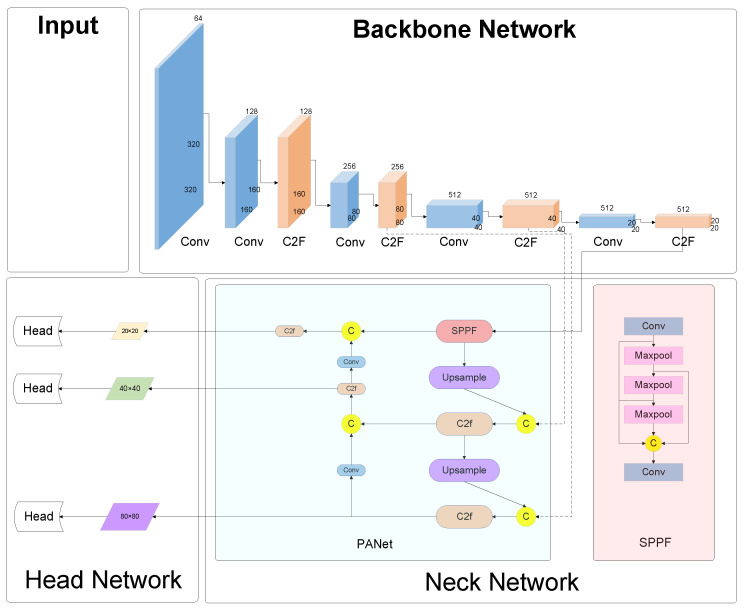
Architecture for YOLOv8 module.

Figure 1 delineates the architecture of the YOLOv8 network, elucidating the sequential stages from image acquisition to detection output. Initially, the input imagery is subjected to selective data augmentation and dimensional adjustments in the preprocessing phase, laying the groundwork for subsequent analyses. This preparatory phase is succeeded by the conveyance of the image to the core backbone network, tasked with the pivotal role of feature extraction.

The essence of this network lies in its neck component, an augmented feature extraction conduit, engineered to amalgamate the extracted attributes. This innovative structure is adept at discerning features across a triad of scales: diminutive (20 × 20), intermediate (40 × 40), and extensive (80 × 80), catering to the nuanced demands of detail across varied object dimensions. The culmination of this process is the integration of these multi-scaled features, which are then meticulously scrutinized by the network’s head. This final stage is instrumental in synthesizing the detection outcomes, encapsulating the essence of the analytical prowess of the YOLOv8 network.

## 3. FasterNet Architecture

Chen et al. [27] proposed the FasterNet in CVPR2023, chasing higher FLOPS for faster neural networks.

In their scholarly endeavor, the nuanced correlation between latency and floating-point operations per second (FLOPs) has been rigorously articulated, as encapsulated within Equation (1). This articulation serves to illuminate the intricate dependencies that govern computational throughput and responsiveness, providing a foundational equation that delineates the interplay between these pivotal metrics.

(1) $$Latency=\frac{Flops}{FLOPS}$$

Within the scope of this manuscript, the term ‘FLOPS’ is employed as a quantifier for computational velocity. Prior investigations have diligently endeavored to curtail FLOPS, albeit with scant regard for the concurrent finesse of FLOPS optimization to attain minimized latency [27].

FasterNet emerges as a novel neural network paradigm, distinguished by its remarkable alacrity and efficacy across a spectrum of visual assignments. This architectural innovation has been achieved through a deliberate simplification process, excising superfluous elements to enhance compatibility with diverse hardware ecosystems.

**Figure 2:**
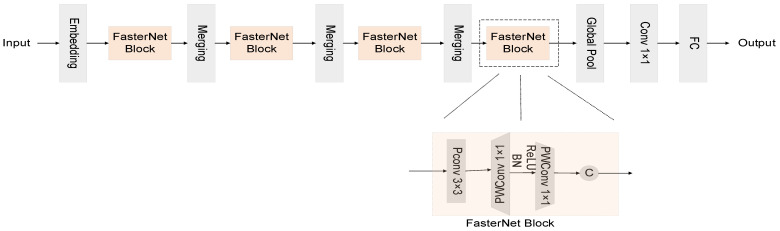
FasterNet’s overall architecture. It consists of four hierarchical layers, each containing a stack of FasterNet blocks, with a preceding embed or fusion layer. Feature classification is performed in the last three layers. Within each FasterNet block, two PWConv levels follow one PConv level.

Figure 2 presents an illustrative depiction of the FasterNet architecture, as elucidated in this discourse. Within the domain of backbone networks, depth-wise convolution, herein referred to as DWConv, stands as a prevalent optimization stratagem. This technique diverges from the traditional convolutional paradigm by allocating a distinct convolution kernel to each channel, thereby eschewing the one-size-fits-all kernel approach. Such a methodology significantly diminishes superfluous computational endeavors and FLOPs, heralding a more efficient computational framework.

Nonetheless, it is pivotal to acknowledge that DWConv, in isolation, does not serve as a panacea, for it bears the potential to compromise the network’s precision. To ameliorate this, DWConv is typically succeeded by Pointwise Convolution (PWConv), a tactical intervention designed to recuperate and augment the network’s precision, thus ensuring a judicious balance between computational efficiency and accuracy.

However, this results in more storage accesses, leading to higher latency and reduced performance. Equation (2) illustrates the storage accesses for DWConv, where h and w represent the length and width of the graph, and c denotes the number of channels [28].

(2) $$h\times w\times 2c^{\prime }+k^{2}\times c^{\prime }\approx h\times w\times 2c^{\prime }$$

For regular convolution, the memory access can be expressed as follows:

(3) $$h\times w\times 2c^{\prime }+k^{2}\times c\approx h\times w\times 2c$$

Observations reveal that the storage access demands of DWConv surpass those associated with the conventional Convolution module. In pursuit of elevating detection capabilities and supplanting the suboptimal performance characteristics of both regular Convolution and DWConv, the inception of a novel convolutional module emerges as an imperative. This innovative module seeks to harmonize efficiency with efficacy, heralding a new epoch in convolutional network design and its application in complex detection tasks.

Diverging from the paradigms of regular Convolution and DWConv, the PConv within FasterNet adopts a more discerning approach by applying regular Convolution to merely a subset of the incoming channels, specifically targeting those from which geospatial functionalities are extrapolated, whilst leaving the remainder of the channels untouched. In instances where the graphical data are stored in a contiguous manner, the initial and terminal contiguous graphs are harnessed to encapsulate the entirety of the graphical information. This methodological pivot to PConv yields a substantial reduction in computational overhead, slashing the requisite number of FLOPs to a mere 1/16th of those demanded by traditional convolutional operations. Moreover, PConv’s design curtails storage access needs to just a quarter of what is typical in regular convolution [28].

**Figure 3:**
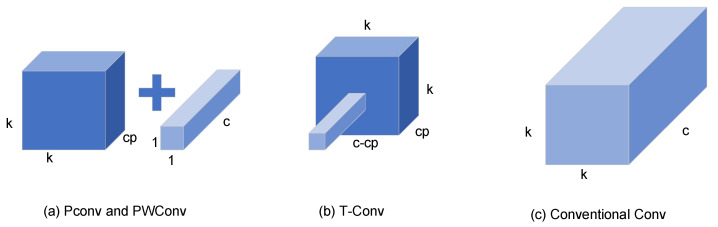
Comparison of various convolution patterns.

To adeptly interlink the correlations pervading the input channels, akin to the strategy employed in DWConv, the FasterNet architecture integrates PWConv in conjunction with PConv. This amalgamation manifests in two distinct configurations: the T-Convolution and a duo of discrete convolutional patterns. Figure 3 offers a concise comparative overview of these convolutional variants, elucidating their distinct characteristics and operational nuances.

T-Convolution distinguishes itself by ascribing enhanced significance to the central position beyond what is customary in standard convolutional operations. This centricity-biased approach facilitates more efficacious computational processes. However, it is noteworthy that this efficiency comes at the cost of an elevated consumption of FLOPs, particularly when juxtaposed against the FLOPs requisites of PConv and PWConv. When maintaining parity in the count of functions both at the ingress and egress, the FLOP dynamics of T-Convolution unfold as follows:

(4) $$h\times w\times (k^{2}\times c_{p}\times c+c\times (c-c_{p}))$$

The FLOPs for PConv and PWConv are, respectively:

(5) $$h\times w\times (k^{2}\times c_{p}^{2}+c\times c_{p})$$

c must be greater than $c_{p}$ and the difference between c and $c_{p}$ must be greater than $c_{p}$, where $c_{p}$ is the number of the 1st or of the last consecutive channel in successive storage accesses. T-convolution FLOPs are greater than PConv and PWConv FLOPs.

As depicted in Figure 3, within each unit of FasterNet, an architectural ensemble comprising one PConv and two PWConv configurations is observed. These units are pivotal in the neural network’s architecture, with normalization and activation layers playing critical roles. Within the ambit of each FasterNet module, the dual PWConv layers are synergistically paired with Batch Normalization (BN) and Rectified Linear Unit (ReLU) layers. The integration of BN not only expedites the training process but also enhances the model’s accuracy. Concurrently, the ReLU layer serves as a catalyst, fostering a swifter learning curve for the model while mitigating the risk of gradient vanishment. Strategically positioned between the PWConv phases in each FasterNet unit, the normalization and activation stages strike a harmonious balance, ensuring both efficiency and the preservation of functional integrity.

## 4. EMA Module

The advent of attention mechanisms marks a significant evolution in the computational landscape, garnering widespread acclaim for their efficacy [29]. The strategic incorporation of such mechanisms is heralded as a promising solution to the quandary of subtle feature detection within diminutive targets, an aspect particularly pivotal in accentuating the relevance of each channel within a computational task [30]. This paradigm ensures that features of paramount importance are accentuated, whilst those of lesser significance are relegated, thereby streamlining the focus towards task-critical information.

Empirical investigations lend substantial credence to the efficacy of both canal and spatial attention mechanisms in cultivating discriminative attributes across a broad spectrum of computer vision pursuits. However, it is crucial to acknowledge that efforts aimed at compressing channel dimensionality, with the intention of elucidating inter-channel interactions, may unintentionally compromise the fidelity of deep visual representations. This delicate balance underscores the need for meticulous consideration in the architectural design of attention mechanisms, ensuring the enhancement of feature discernment without detracting from the foundational visual constructs.

In this context, a novel module named Efficient Multi-Scale Attention (EMA), as proposed by D. Ouyang [31] and colleagues, emerges as a salient innovation. This module is designed to preserve the sanctity of channel information while simultaneously curtailing computational demands. By reconfiguring multiple channels to encapsulate stack measurements and amalgamating channel metrics into a constellation of partial features, EMA endeavors to refine the temporal distribution of features across various characteristic groups, thus optimizing the overall feature landscape.

**Figure 4:**
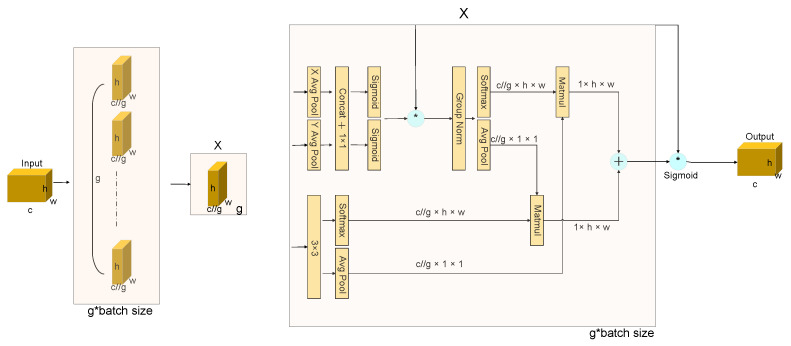
EMA module. Here * means the process of re-weight.

The EMA module, as depicted in Figure 4, adopts a parallel processing paradigm to circumvent the pitfalls of protracted sequential computation, thereby facilitating enhanced depth within the network’s architecture. This innovative approach allows EMA to adeptly capture channel-specific nuances through convolutional maneuvers, all the while maintaining the original dimensionality. This strategy ensures the provision of refined pixel-level attention across high-resolution feature maps, thereby elevating the quality of the extracted features.

Central to the EMA module’s operational efficacy is the synergistic use of a 3 × 3 kernel coupled with a 1 × 1 branch. This combination is strategically employed to assimilate multi-scale spatial information, engendering a swift and efficient response mechanism. This architectural nuance enables the EMA module to adeptly navigate the complex landscape of feature extraction, ensuring a robust and dynamic adaptation to varying spatial scales within the visual data.

Grouping of Features. For any given input characteristic diagram $X\in R^{C\times H\times W}$, in the channel dimension, EMA will divide X into G sub-features. This paper allows different semantics to be learnt, grouping style defined as $X=\left[X_{0},X_{i},\ldots ,X_{G-1}\right],X_{i}\in R^{C//G\times H\times W}$. Without losing generality, the paper assumes that G≪C and the image processing of the area of interest in each subsample is enhanced by the learned attention weight descriptors.

Parallel Subnetworks. Neurons, endowed with expansive local receptive fields, are adept at assimilating multiscale spatial data. The EMA framework proposes a tripartite parallel pathway for the derivation of attention weights from the aggregated maps, with two pathways residing within the 1 × 1 bifurcation and the third within the 3 × 3 bifurcation. This architectural construct facilitates a nuanced modeling of cross-channel informational exchanges along the channel dimension, thereby enabling the capture of dependencies that span the entire channel spectrum while simultaneously optimizing computational efficiency. To further enhance the model’s capability to delineate multi-scale feature descriptors, a 3 × 3 kernel is strategically superposed within the 3 × 3 manifold. This addition serves to augment the model’s perceptual depth, enabling a more comprehensive and nuanced feature representation. Through this sophisticated interplay of convolutional dynamics and attention mechanisms, the model achieves a heightened proficiency in feature discernment, pivotal for tasks requiring intricate spatial awareness and feature granularity.

The G group undergoes reshaping and relocation within the batch dimension, while the input tensor is redefined in the shape of C//G×H×W. The G group will be converted to the batch size. The 1 × 1 convolution output is split into two separate convolution vectors, and two non-linear sigmoid operators are used to apply a 2-dimensional two-norm deviation to the linear convolution. On the other hand, the 3 × 3 fork uses a 3 × 3 transform to preserve the regional connections between channels and increase the characteristic space. This allows EMA to preserve precise spatial structure information within channels while capturing inter-channel detail and adjusting the importance of individual channels.

Cross-Spatial Learning. An approach is proposed to aggregate rich features using spatial information aggregation with different spatial orientations. The method involves introducing two different data tensors: one being the exit of the 1 × 1 bifurcation and the other of the 3 × 3 bifurcation. In addition, the output of the minimum bifurcation is transformed into the appropriate scale form before the channel characteristics are activated together, i.e., $R_{1}^{1\times C//G}\times R_{3}^{C//G\times HW}$. The 2D global pooling operation is presented as Equation (6).

(6) $$z_{c}=\frac{1}{H\times W}\sum_{j}^{H}\sum_{i}^{W}x_{c}\left(i,j\right)$$

Encoding global information and modelling remote dependencies is the goal of this design. The result of the parallel processing described above is then multiplied by the dot product operation of the matrix, resulting in the first spatial attention map. The 1 × 1 twigs are directly transformed into the appropriate forms, i.e., $R_{3}^{1\times C//G}\times R_{1}^{C//G\times HW}$. This methodology sets the stage for the activation mechanism of joint channel features. Subsequently, a secondary spatial attention map is forged, meticulously conserving the entirety of spatial positional information. The culmination of this process involves the amalgamation of the duo of spatial attention weights produced, followed by the application of a sigmoid function to derive the output feature map for each respective group. This procedural flow accentuates the holistic context enveloping all pixels, concurrently collating the pairwise interrelations at the pixel granularity.

The outcome engendered by the EMA module retains dimensional parity with X, ensuring seamless integrability and operational efficiency within contemporary system architectures. This attribute underscores the module’s capability to enhance feature representation without imposing additional spatial burdens, thereby rendering it an invaluable asset in the landscape of modern computational frameworks.

The EMA module strategically allocates attention by aligning descriptors of global and local features, enabling the modeling of long-range dependencies and incorporating accurate positional data through cross-space aggregation. This enhances the EMA’s ability to generate detailed contextual insights, improving attention granularity in high-resolution attribute maps.

The adoption of CNNs plays a pivotal role in this context, leveraging their prowess to amalgamate contextual information across diverse scales. This fusion process is instrumental in augmenting the attention mechanism, ensuring a nuanced understanding of feature interrelations. The subsequent parallelization of convolution cores emerges as an efficacious strategy, adeptly navigating the complex terrain of both short and long-range interactions through deep learning methodologies.

This parallel deployment of 3 × 3 and 1 × 1 convolutions significantly amplifies the capacity to capture mutual information among interacting elements, starkly contrasting with the incremental responses characteristic of bounded perceptual fields. Such an architectural innovation ensures a more comprehensive and dynamic comprehension of the visual domain, markedly enhancing the efficacy of attention-based feature extraction.

## 5. Method

Underwater target detection technology plays a pivotal role in the realm of oceanic exploration. Nevertheless, the complexity of the underwater environment and the presence of numerous minuscule targets often impedes the efficacy of existing detection systems. These systems typically fall short of desired performance benchmarks and possess large model sizes, rendering them unsuitable for deployment on ROV with stringent memory constraints. To address these challenges, we have refined and developed a real-time underwater target detection model based on YOLOv8, which surpasses current technologies in both detection speed and accuracy. Specifically, the model includes backbone and neck layers optimized for underwater characteristics as well as C2f modules. This enhanced version of the YOLOv8 algorithm significantly improves the detection performance for small underwater objects, achieving high accuracy and meeting the speed requirements for real-time detection. Moreover, the optimized model is characterized by a compact weight file and reduced computational resource demands, facilitating its seamless integration into lightweight detection systems powered by underwater wireless sensors.

### 5.1. Improved C2f Modules

**Figure 5:**
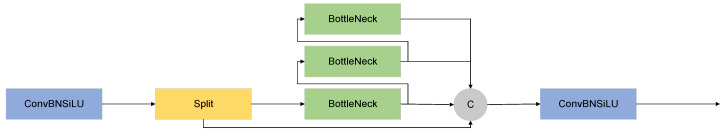
C2f module.

**Figure 6:**
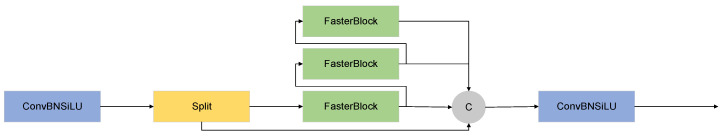
C2f-Faster module.

In tackling the constraints imposed by the memory capacity of the piggyback platforms utilized in underwater target detection, it is imperative for the detection algorithm to navigate the fine line between precision and compactness. This dissertation contributes to the enhancement of the YOLOv8 model’s C2f component. Figure 5 and Figure 6 illustrate the original C2f model alongside its evolved counterpart, the C2f-Faster model, showcasing the advancements made in refining the model’s efficiency without compromising its effectiveness.

Through the integration of more refined computational algorithms and optimization tactics, the inference velocity of the model has seen substantial enhancement. This evolution stands as particularly beneficial in the realm of real-time engagements or in situations that demand an elevated throughput capability. Noteworthy is the advent of C2f-Faster, which bolsters the model’s precision without veering from its core architectural principles. This implies that, under identical conditions of data and task specificity, C2f-Faster is equipped to surpass its predecessors in performance metrics.

The evolution of the C2f model into C2f-Faster marks a significant stride towards adaptability across a diverse spectrum of data types and complexities. This progression augments the model’s proficiency in generalizing across novel and uncharted datasets, thereby fortifying its robustness and dependability.

Furthermore, C2f-Faster contributes to a reduction in the model’s demands on computational and memory resources by streamlining these aspects. This efficiency not only facilitates the model’s scalability across larger datasets and more intricate tasks, but also enhances its overall utility in a broad array of applications.

### 5.2. Efficient Attention Mechanism

In order to address the challenges of reduced small-scale object localization information and reduced sampling efficiency due to increased network depth, we incorporate the EMA attention mechanism.

**Figure 7:**
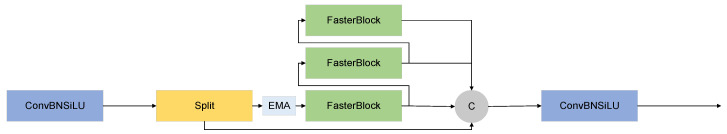
C2f-Faster-EMA module.

Figure 7 illustrates the substantial refinements made to the C2f-Faster model, culminating in a notable uplift in its operational efficacy and an augmented proficiency in the detection of diminutive targets. The advent of the enhanced C2f-Faster-EMA model heralds a suite of potential advantages, primed to significantly bolster the model’s perceptual acuity. This enhancement is primarily attributed to the integration of multi-scale feature fusion alongside sophisticated attention mechanisms, which collectively empower the model to adeptly assimilate contextual nuances and the multifaceted characteristics of targets across varying scales.

The ramifications of these technological advancements are multifarious, promising a marked elevation in the precision and dependability of target detection. By virtue of its refined ability to discern and localize targets through the recognition of multi-scale features, the model stands poised to redefine benchmarks in target detection and localization. This evolution in model capabilities underscores a significant leap forward in the realm of computational perception, setting new paradigms in the accuracy and reliability of target detection endeavors.

### 5.3. Improved Backbone and Neck Layers

**Figure 8:**
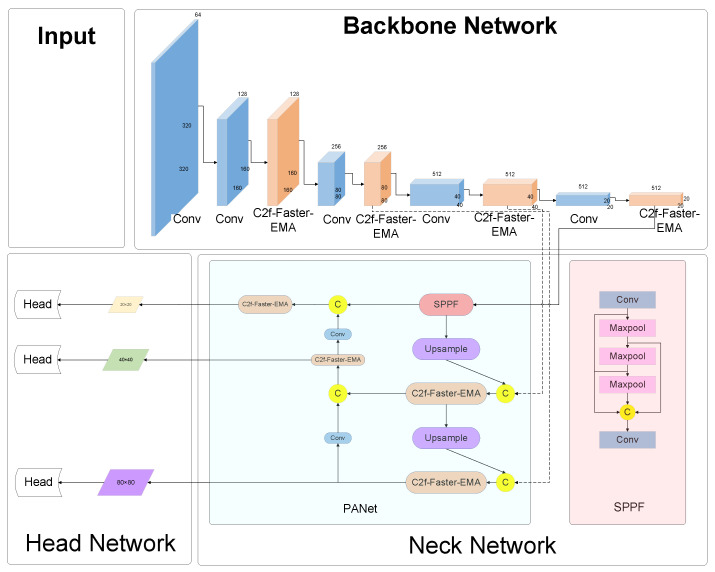
Architecture for the YOLOv8-C2f-Faster-EMA module.

Figure 8 illuminates the evolved network architecture, a direct consequence of the aforementioned enhancements. Stemming from this refined design, two variant configurations have been conceived, each tailored to accommodate the diverse potential placements of the C2f-Faster-EMA modules within the network. These adaptations are delineated as follows, offering a glimpse into the architectural flexibility and the strategic positioning of EMA modules to optimize the network’s performance across varying computational paradigms and application scenarios.

(1) YOLOv8-C2f-Faster-EMA: This iteration marks a significant evolution, wherein the C2f-Faster-EMA module universally supersedes the C2f components throughout the network’s expanse. This uniform adoption of the EMA-enhanced module signifies a holistic upgrade, promising a synergistic boost in the network’s efficiency and efficacy.

(2) YOLOv8-C2f-Faster-EMAv2: Characterized by targeted augmentation, this variant strategically positions the C2f-Faster-EMA module within the neck segment, supplanting the conventional C2f modules. This focused enhancement aims to leverage the EMA’s strengths in a critical area of feature processing, while the backbone remains anchored by the steadfast C2f-Faster modules, ensuring robust foundational support.

(3) YOLOv8-C2f-Faster-EMAv3: Distinguished by its architectural refinement, this version sees the integration of the C2f-Faster-EMA module within the network’s backbone, effectively phasing out the traditional C2f elements. Concurrently, the neck region retains the C2f-Faster module, fostering an equilibrium between cutting-edge enhancement and structural fidelity.

## 6. Experiments

### 6.1. Experiment Introduction

This section first introduces the dataset used in this paper, then introduces the experimental environment and training strategy, and, finally, introduces the evaluation metrics related to the experimental results.

#### 6.1.1. Dataset

The Trash_ICRA19 dataset emerges as a pivotal open-source compendium for the identification of submerged entities within the marine sphere, meticulously annotated following the PASCAL VOC dataset framework. This repository is delineated into three principal classifications: plastic, biological matter, and remotely operated vehicles (ROVs). The training corpus encompasses 5720 optical submarine images, while the validation segment comprises 820 images of a similar nature. The dataset designated for testing includes 1144 optical submarine photographs in JPEG format.

**Figure 9:**
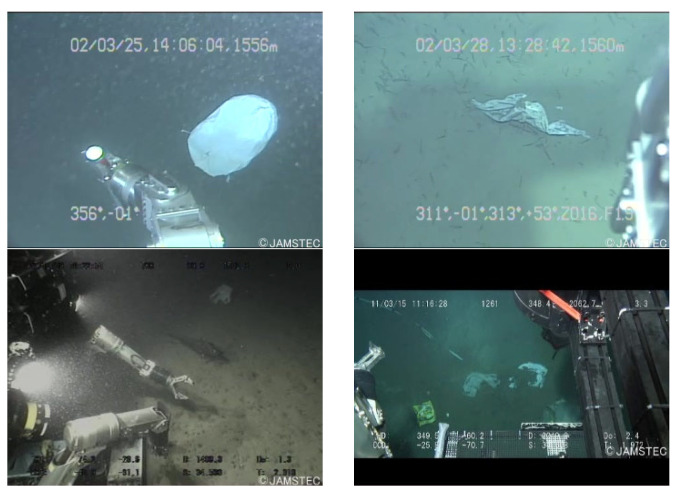
Sample of partial datasets.

Delving into the specifics, the validation subset is enriched with 853 instances of plastic debris, 70 biological specimens, and 141 ROV units. In parallel, the testing array presents a composition of 937 plastic items, 396 biological entities, and 335 ROVs, offering a comprehensive spectrum for analytical pursuits. For illustrative clarity, Figure 9 curates a select excerpt from this dataset, providing a visual gateway into the diverse range of submerged objects it encompasses.

#### 6.1.2. Experimental Environment and Training Strategies

The hardware platform and environmental parameters used in the experimental training phase are shown in Table 1.

Some of the key parameter settings during model training are shown in Table 2.

Moreover, in our experiments, the ‘close_mosaic’ parameter was set to 10, a strategic choice yielding several benefits:

1. Mitigation of Overreliance on Data Augmentation: Although the Mosaic method substantially enhances data diversity, excessive reliance on it can lead the model to learn non-realistic image characteristics. Setting ‘close_mosaic’ to 10 implies that, towards the end of the training process (such as the final 10 epochs), we cease using Mosaic data augmentation, allowing the model to fine-tune its performance under more conventional image conditions.
2. Simulation of a More Realistic Application Environment: Reducing or halting Mosaic data augmentation in the final training phase aids the model in better adapting to actual image conditions, thereby enhancing its accuracy and robustness in real-world settings.
3. Balancing Training Efficiency and Resource Consumption: Data augmentation, especially complex methods like Mosaic, can prolong the training time per epoch. Diminishing reliance on these methods towards the end of the training helps to reduce additional time and resource expenditure once the model has already acquired sufficient features.
4. Optimization of Model Performance: In the terminal phase of training, typically a period of ‘refinement’ or ‘fine-tuning’ occurs, where the focus shifts to optimizing the model’s adaptability to the existing data, rather than continuing to feed it with a vast array of highly varied augmented data.

#### 6.1.3. Evaluation Indicators

In this study, we employed mAP, R, and P to quantitatively assess the efficacy of our proposed methodologies [32]. The mAP, a benchmark for evaluating object detection algorithms, was calculated using the DOTA metric. Precision reflects the model’s capability to correctly identify relevant objects, representing the fraction of correctly predicted objects among all predictions made by the model. Recall measures the model’s capacity to identify all pertinent objects, indicated by the maximum number of true objects that the model’s predictions can encompass. The calculations for P, R, and AP were conducted as follows:

(7) $$P=\frac{T_{p}}{T_{P}+F_{P}}\times 100\%$$

(8) $$R=\frac{T_{P}}{T_{P}+F_{N}}\times 100\%$$

(9) $$AP=\int_{0}^{1}P\left(R\right)dR$$

where $T_{P}$ is true positive, $F_{P}$ is false positive, and $F_{N}$ is false negative.

### 6.2. Experiment Results

#### 6.2.1. Effect of FasterNet

In this comparative analysis, we scrutinized the computational efficiency and performance metrics of various YOLOv8 model iterations, including the baseline and its derivatives featuring different architectural enhancements. UAV platforms have limited resources, making it hard to embed high computational and storage-demanding object detection models [33]. The evaluation metrics encompassed Giga Floating Point Operations per Second (GFLOPs), latency measured in milliseconds (ms), total processing time for a standard dataset in hours (h), and frames per second (FPS). Nonetheless, it is important to note that greater accuracy does not always correlate with improved efficiency, particularly in terms of scalability and speed [34].

As shown in Table 3, the baseline YOLOv8 model, with 8.1 GFLOPs, demonstrated a commendable balance between computational load and processing speed, achieving a latency of 0.23 ms and an operational efficiency of 109.7 FPS over a duration of 3.475 h. The introduction of the ‘fasternet’ augmentation resulted in an increase in GFLOPs to 10.7, indicating a higher computational demand, which correspondingly led to a slight increase in latency to 0.30 ms and a marginal decrease in processing speed to 98.3 FPS, extending the total processing time to 3.766 h.

In contrast, the YOLOv8-C2f-Faster variant exhibited a reduction in GFLOPs to 6.4, reflecting a decrease in computational complexity. This model achieved the lowest latency of 0.18 ms and a notable increase in efficiency to 125.5 FPS, thereby reducing the overall processing time to 3.165 h. The subsequent iterations, YOLOv8-C2f-Faster-EMA and its versions (v2 and v3), maintained a similar range of GFLOPs (6.5–6.6), with marginal variations in latency (0.18–0.19 ms). Notably, these models demonstrated a progressive improvement in processing speed, culminating in the YOLOv8-C2f-Faster-EMAv3 achieving the highest efficiency of 129.3 FPS and the shortest processing time of 3.042 h.

These findings highlight the nuanced impact of architectural modifications on the computational efficiency and performance of object detection models. The YOLOv8-C2f-Faster-EMAv3 iteration emerges as the epitome of efficiency, striking a harmonious equilibrium between computational demands and processing alacrity. This balance significantly elevates the potential of our model for deployment in real-time object detection tasks, particularly in the nuanced domain of underwater refuse identification, where it proves exceptionally viable on platforms constrained by limited computational resources.

#### 6.2.2. Effect of EMA

In this comprehensive evaluation, we examined the performance enhancements across various iterations of the YOLOv8 model, as summarized in Table 4. The assessment focused on key metrics including Precision, Recall, mean Average Precision at an Intersection over Union (IoU) threshold of 50% (mAP@50), and mAP across IoU thresholds ranging from 50% to 95% (mAP@50:95).

The baseline YOLOv8 model established a foundational benchmark with a Precision of 72.5%, Recall of 75.7%, a mAP@50 of 79.6%, and a mAP@50:95 of 53.2%. The introduction of the ‘fasternet’ adaptation in YOLOv8-fasternet resulted in marginal improvements in Precision (73.5%) and Recall (78.9%), alongside an increase in mAP@50 to 81.9%, albeit with a slight decrease in mAP@50:95 to 52.8%. The YOLOv8-C2f-Faster variant, despite a reduction in Precision to 70.5% and Recall to 74.6%, achieved a mAP@50 of 80.2%. However, it exhibited a notable decrease in mAP@50:95 to 48.2%, suggesting a trade-off between computational efficiency and detection accuracy across a wider range of IoU thresholds.

To enhance the precision of the model, we incorporated the EMA attention mechanism into the C2f-Faster module, giving rise to the YOLOv8-C2f-Faster-EMA construct. Nonetheless, the deployment of this module across the backbone, neck, and head layers, while localizing information predominantly within the backbone, inadvertently resulted in a disproportionate focus on regions of minimal significance. This skewed emphasis detrimentally impacted the model’s proficiency in assimilating pertinent information. In the YOLOv8-C2f-Faster-EMAv3 architecture, the attention mechanism is confined to the backbone layer, a strategic decision that enables the model to hone in on critical local information while minimizing focus on extraneous areas, thereby augmenting its efficacy. Attention mechanisms, by their nature, entail considerable computational demands, necessitating extensive memory and storage to ascertain correlations across all input locations and to manage the resultant data. By limiting the scope of the attention mechanism to the backbone layer, there is a notable reduction in the computational load, enhancing the model’s efficiency and diminishing the requirements for storage and memory. This approach not only streamlines the model’s operational demands but also simplifies its implementation and utilization, offering a more streamlined and user-friendly experience.

Notably, the YOLOv8-C2f-Faster-EMAv3 iteration marked a significant leap in performance, achieving the highest Precision (79.2%) and Recall (79.8%) among the models evaluated. This version also set new benchmarks for mAP@50 and mAP@50:95, at 84.6% and 55%, respectively, indicating a substantial enhancement in both accuracy and consistency across varying IoU thresholds.

These findings elucidate the intricate balance between architectural modifications and performance metrics in object detection models. The YOLOv8-C2f-Faster-EMAv3 stands out as the most advanced iteration, offering a compelling blend of P, R, and AP, thereby underscoring its efficacy for diverse object detection applications.

#### 6.2.3. Effect of Mosaic

**Figure 10:**
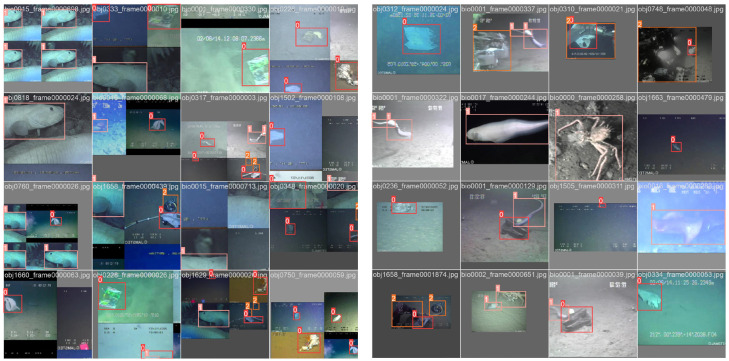
Comparison of batch-size before and after the addition of the Mosaic algorithm.

Figure 10 delineates the alteration in batch-size subsequent to the incorporation of the Mosaic algorithm for network training. Initially, a batch-size of 16 signifies the GPU’s capability to process 16 images concurrently. However, with the implementation of the Mosaic algorithm, this capacity escalates, allowing the GPU to handle 64 images simultaneously. This enhancement significantly amplifies the training efficiency of the algorithm, demonstrating the algorithm’s augmented computational throughput and efficacy in handling larger data sets in parallel.

The comparative experiment in Table 5 shows that using the mosaic module improves the model’s precision, recall, and mAP at 50% IoU. Specifically, precision jumps from 72.8% to 79.2%, recall from 76% to 79.8%, and mAP from 82.4% to 84.6%, indicating that the mosaic module leads to more accurate object detection.

#### 6.2.4. Comparative Experiment

To substantiate the superiority of the refined model, this study conducted a series of benchmarking experiments, juxtaposing its performance with that of prevalent object tracking systems. These systems encompass the computationally intensive Faster R-CNN, the efficient SSD, the compact yet potent YOLOv7-tiny, and the more advanced YOLOv7. Moreover, the investigation extended to recently proposed and widely discussed models within the scholarly community, namely YOLOv8-goldyolo, YOLOv8-convnextv2, YOLOv8-swintransformer, and YOLOv8-vanillanet, each embodying cutting-edge developments in the realm of object detection.

Table 6 presents a succinct comparison of various object detection models, contrasting computational load and detection performance. The Faster R-CNN model, while computationally intensive, shows high Recall but lower Precision, resulting in a moderate mAP@50. The SSD model offers a better balance of Precision and GFLOPs but with lower Recall.

The YOLOv7 variants, particularly YOLOv7-tiny, demonstrate an impressive trade-off between efficiency and accuracy, achieving high mAP@50 with minimal computational demand. YOLOv8 variations, with a focus on architectural diversity, exhibit varied trade-offs between Precision, Recall, and GFLOPs.

The authors’ model stands out with its exceptional mAP@50 and high Recall at the lowest GFLOPs, showcasing a model that is both computationally efficient and effective in object detection, thus representing a significant advancement in the field.

In this study, we sought to enhance the model’s predictive capabilities by integrating alternative attention modules in place of the EMA Attention module, while keeping the C2f-Faster module unchanged. To this end, we assessed the efficacy of three distinct attention mechanisms: SE (Squeeze and Excitation) [35], CA (Coordinated Attention) [36], and ECA (Efficient Channel Attention) [37], as delineated in this manuscript.

The empirical findings, presented in Table 7, indicate that within the context of underwater debris identification utilizing the C2f-Faster module, the EMA attention schema surpasses the SE, CA, and ECA mechanisms in terms of mAP@0.5 metrics. Furthermore, the network adeptly leverages the synergies between the Faster Block and EMA to furnish effective channel representations during convolutional operations, without diminishing channel dimensions. This synergy enhances attentional efficacy, culminating in superior detection precision. These observations underscore the EMA attention mechanism’s aptness for the envisaged model, culminating in augmented accuracy in object detection and localization.

#### 6.2.5. Result in Terms of Target Detection on the Trash_ICRA19 Dataset

As shown in Table 8, in the realm of biological detection, YOLOv8 presents initial benchmarks with Precision, Recall, and mAP@50 at modest levels of 1.62%, 1.43%, and 1.65%, respectively. In stark contrast, our refined model markedly transcends these figures, achieving Precision and Recall of 6.92% and 8.57%, alongside a mAP@50 of 3.19%. This pronounced improvement underscores our model’s enhanced sensitivity and specificity in detecting biological features even with a small sample size.

Focusing on ROV detection, YOLOv8 achieves a Precision of 62.8% and Recall of 52.5%, culminating in a mAP@50 of 53.8%. Our model, however, excels beyond these parameters, registering a Precision of 74.4%, a Recall of 56%, and a mAP@50 of 55.3%, thereby evidencing its superior acumen in discerning ROV attributes.

When evaluating across a holistic dataset that amalgamates all elements, YOLOv8 secures a Precision of 49.5%, Recall of 44.4%, and mAP@50 of 45.5%. Our model slightly refines Precision to 49.6% and more significantly enhances Recall to 47% and mAP@50 to 47.2%, indicating a more balanced and robust performance across a diverse array of detection challenges.

This analytical overview accentuates the bespoke capabilities of our model, particularly in its refined detection of biological components and ROV elements, heralding its versatility and potential applicability across a broad spectrum of marine object detection scenarios.

#### 6.2.6. Result on the TrashCan Dataset

To substantiate the enhanced efficacy of our refined model in trash detection, we undertook an additional evaluation employing the TrashCan dataset.

The TrashCan dataset, consisting of 7212 annotated images, showcases marine trash, ROVs, and diverse undersea life, utilizing detailed instance segmentation for precise object identification. Originating from the J-EDI (JAMSTEC E-Library of Deep-sea Images) dataset by the Japan Agency of Marine Earth Science and Technology (JAMSTEC), it includes decades of ROV video data from the Sea of Japan. With two versions, TrashCan-Material and TrashCan-Instance, tailored for different classification needs, this dataset aims to advance trash detection methods for marine robotics. Notably, TrashCan stands out as possibly the first underwater trash dataset with instance-segmentation annotations, poised to drive forward research in autonomous marine trash detection and removal.

Table 9 showcases the performance of different YOLOv8 iterations, highlighting their Precision, Recall, and mAP@50. The baseline YOLOv8 demonstrates a solid start with balanced metrics. Subsequent versions like YOLOv8-fasternet and YOLOv8-C2f-Faster offer variations in Precision and Recall, aiming for balanced detection capabilities. The YOLOv8-C2f-Faster-EMA focuses more on Recall, while YOLOv8-C2f-Faster-EMAv2 tilts towards Precision. Notably, YOLOv8-C2f-Faster-EMAv3 excels with the highest Precision and a notable improvement in mAP@50, showcasing significant advancements in detection accuracy.

This progression underscores the tailored enhancements in the YOLOv8 series, particularly with the YOLOv8-C2f-Faster-EMAv3, indicating a strategic improvement in accuracy and efficiency. These developments hint at the model’s generalizability and potential for broader applications in remote sensing and object detection fields.

## 7. Discussion

### 7.1. Comparison of Different Models

**Figure 11:**
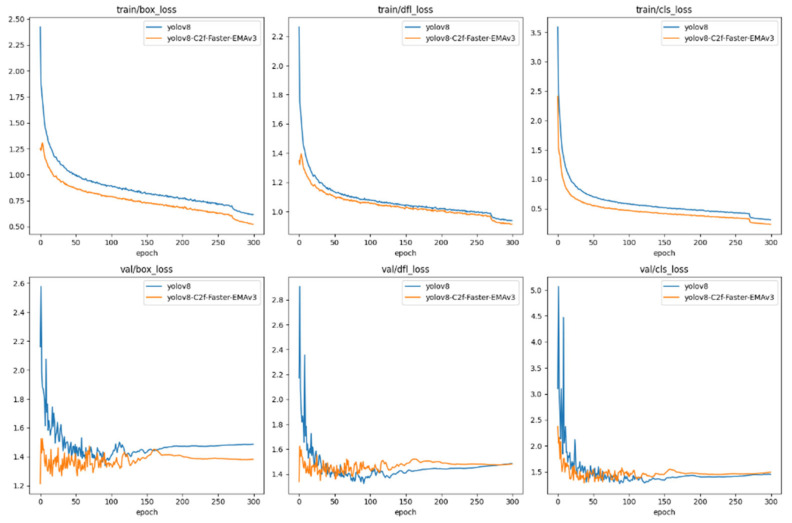
Loss changes of each model.

As delineated in Figure 11, the loss trajectories articulate the comparative performance of two object detection models: the canonical YOLOv8 and its augmented counterpart, YOLOv8-C2f-Faster-EMAv3, across the spectrum of training and validation phases. These phases interrogate different loss dimensions: bounding box accuracy, object detection confidence (‘df1’), and category classification (‘cls’).

The descent in loss values observed in both models is emblematic of the learning process, with YOLOv8-C2f-Faster-EMAv3 manifesting a more precipitous initial decline. This is indicative of an expedited proficiency in the accuracy of bounding box localization. The box loss, a measure of the model’s prowess in affirming object presence, plummets significantly for the YOLOv8-C2f-Faster-EMAv3 variant, underscoring its heightened acuity in object discernment. Similarly, the classification loss trends affirm the YOLOv8-C2f-Faster-EMAv3′s superior capacity for early and precise category identification, maintaining a diminished loss relative to the baseline model.

In the validation arena, the YOLOv8-C2f-Faster-EMAv3 sustains a consistently lower loss in bounding box precision compared to the baseline model, intimating a more robust generalization to novel data samples. This trend is paralleled in the object loss domain, with the YOLOv8-C2f-Faster-EMAv3 variant maintaining a reduced loss, potentially signifying a greater fidelity in predicting true positives. The classification loss in the validation phase corroborates the model’s efficacy in not just learning but also generalizing class distinctions to new exemplars.

Collectively, the YOLOv8-C2f-Faster-EMAv3 transcends the foundational YOLOv8 across all facets of loss evaluation during both training and validation. The pronounced decline and sustained lower plateaus of the loss curves in the YOLOv8-C2f-Faster-EMAv3 are testaments to the beneficial integration of the EMAv3 attention mechanism and the attendant architectural enhancements. Such refinements have yielded a model of increased robustness, adept at striking an equilibrium between localizing, discerning, and classifying objects within both familiar and unseen datasets. The consistent and lower loss valuations in the validation phase also hint at the model’s diminished susceptibility to overfitting and its enhanced capacity for generalization, a quintessential attribute for real-world object detection applications.

**Figure 12:**
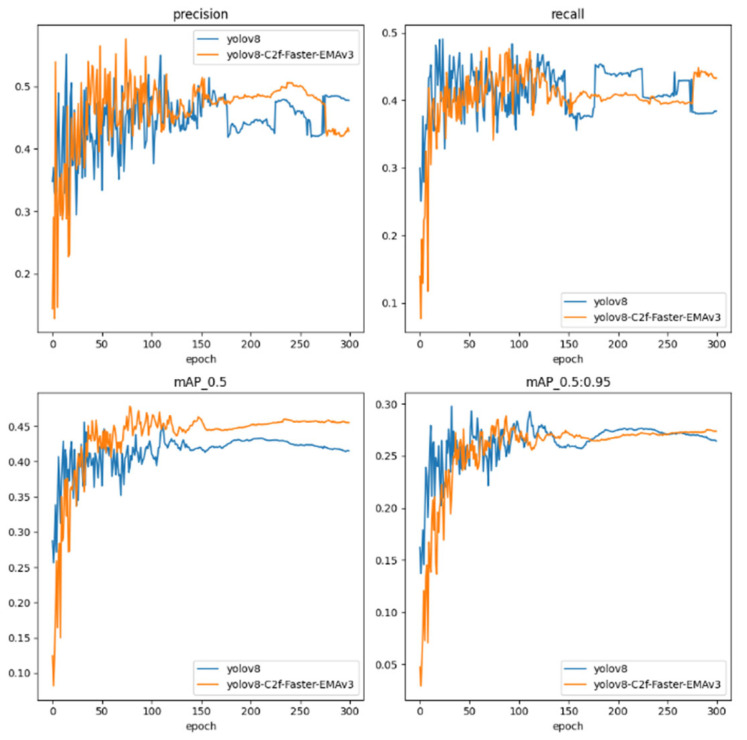
Changes in the four indicators of each mode.

As shown in Figure 12, we conduct a comparative analysis between the archetypal YOLOv8 framework and its architecturally enhanced iteration, the YOLOv8-C2f-Faster-EMAv3. This analysis traverses the training epoch continuum, delineating the models’ performance on pivotal metrics such as Precision, Recall, mean Average Precision at an Intersection over Union (IoU) threshold of 0.5 (mAP_0.5), and mean Average Precision spanning a spectrum of IoU thresholds from 0.5 to 0.95 (mAP_0.5:0.95).

The Precision trajectory, a gauge of the veracity of positive detections, reveals the YOLOv8 model’s fluctuating yet generally stable trend. In contrast, the YOLOv8-C2f-Faster-EMAv3 evidences an ameliorated Precision, particularly manifest in the latter epochs, signaling its honed proficiency in rendering accurate detections.

Turning to Recall, a measure of the model’s comprehensiveness in identifying pertinent instances within the dataset, we observe a parity in performance between the models, punctuated by considerable volatility. Notably, the YOLOv8-C2f-Faster-EMAv3 intermittently outpaces the baseline, intimating at its fortified capability in ensnaring a complete set of object instances.

The mAP_0.5 metric, evaluating the harmonization of precision and recall at an IoU threshold of 0.5, sees the YOLOv8-C2f-Faster-EMAv3 ascend to a more pronounced elevation relative to its YOLOv8 counterpart, indicative of its enhanced detection acumen at this discrete threshold.

In the rigorous assessment of mAP_0.5:0.95, spanning a gamut of IoU thresholds, both models ride a trajectory of high variability. Yet, the YOLOv8-C2f-Faster-EMAv3 sporadically notches higher values, alluding to its adeptness in sustaining detection efficacy across a diversified range of IoU benchmarks.

Perusing the quartet of performance metrics, the YOLOv8-C2f-Faster-EMAv3 displays a propensity for augmented performance, notwithstanding the inherent variability characteristic of the training cadence. This model crystallizes its gains predominantly in the realm of Precision and, albeit less consistently, in Recall and mAP metrics. Such observations suggest that the architectural refinements integrated within the YOLOv8-C2f-Faster-EMAv3 endow it with a preeminent capacity to discern and categorize objects accurately under a breadth of conditions, yet they also reveal avenues for further stabilization and consistency enhancement.

These metrics serve as quintessential barometers of the robustness and dependability of object detection models, especially in scenarios where precision and comprehensive detection are paramount. The depicted trends confer upon the YOLOv8-C2f-Faster-EMAv3 the potential to operate efficaciously across a range of demanding and heterogeneous detection environments, positing it as an auspicious contender for deployment in real-world applications that requisition high fidelity in object detection.

### 7.2. Effect Diagram

**Figure 13:**
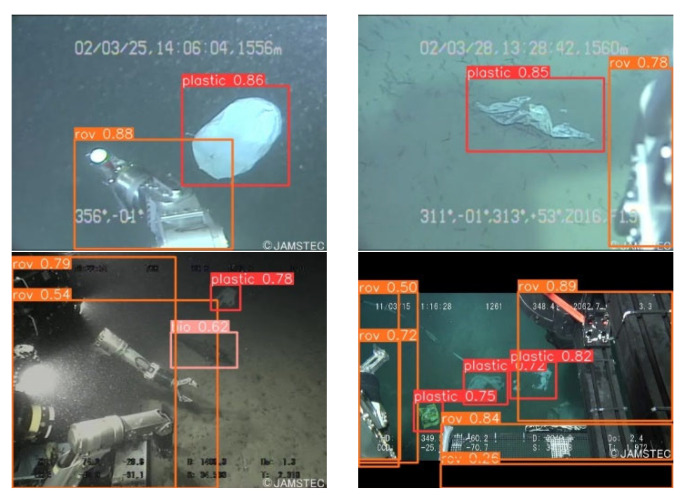
Test result graphs of YOLOv8n.

**Figure 14:**
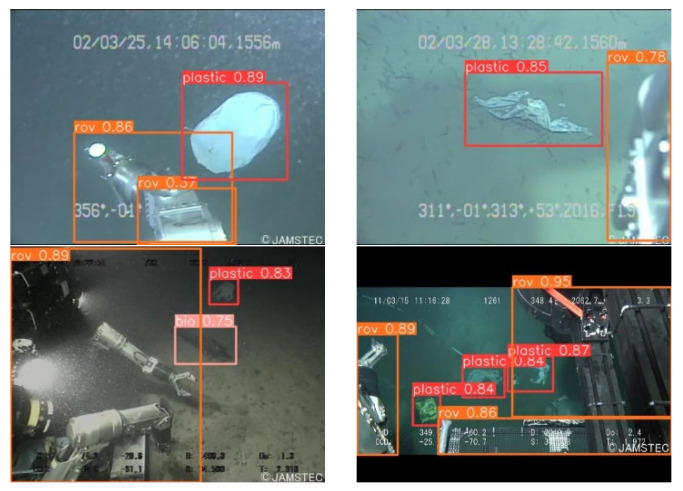
Test result graphs of YOLOv8-C2f-Faster-EMAv3.

Figure 13 and Figure 14 encapsulate the empirical evidence of the model’s adeptness in the identification of underwater refuse, a task critical for environmental monitoring. Within the ambit of well-illuminated conditions, the model manifests a detection precision approximating 0.89 for such debris. This marks a substantial refinement over the foundational YOLOv8n algorithm. Moreover, this enhanced detection capability is not compromised under the exigencies of low-light environments, where it continues to eclipse the performance of the original YOLOv8.

The schematics presented in these figures exhibit a heightened degree of delineation, reflecting the algorithmic advancements achieved. The YOLOv8-C2f-Faster-EMAv3, through its algorithmic innovations, exhibits a pronounced efficacy in not only discerning underwater trash but also in classifying other diminutive targets with high fidelity.

This methodological enhancement effectively mitigates the challenges often associated with missed and spurious detections, thus bestowing upon the network a heightened acuity in detection accuracy. The implications of such advancements are profound, offering promising avenues for enhancing the precision of ecological assessments in marine environments.

**Figure 15:**
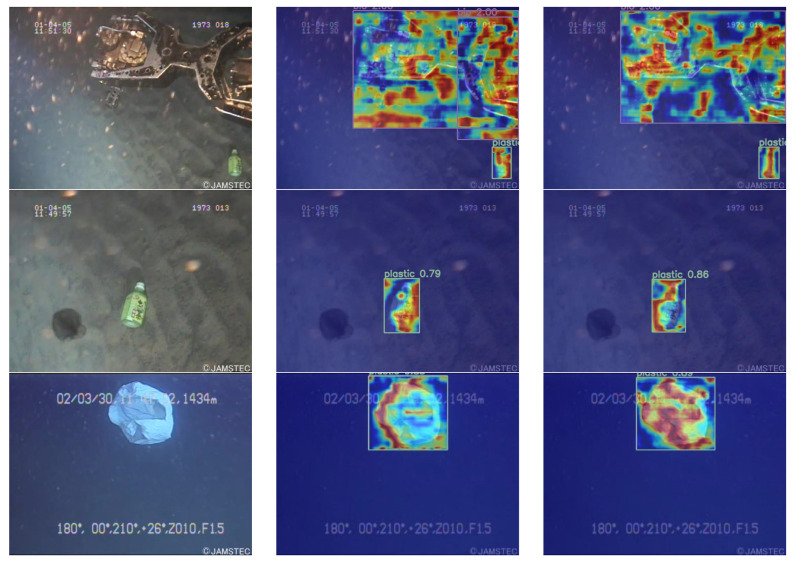

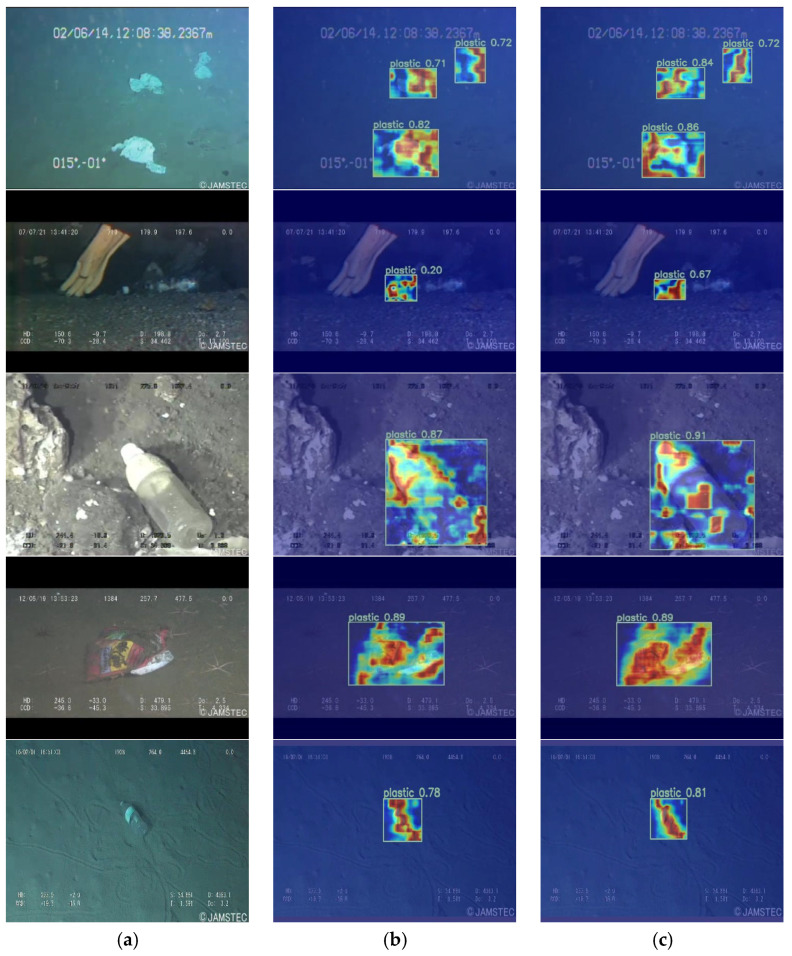
Comparison of heat maps of different network models. (**a**) Original figure; (**b**) YOLOv8n heat map; (**c**) YOLOv8-C2f-Faster-EMAv3 heat map.

Figure 15 presents a series of heat maps comparing the original YOLOv8n (b), and the enhanced model (c). These visual representations elucidate the superior performance of the proposed model over its predecessors. Notably, the introduction of an attention mechanism in the modified model markedly augments its acuity in detecting diminutive targets, thereby substantially elevating its proficiency in distinguishing spurious elements from genuine objects of interest. This strategic enhancement in the model’s architecture underscores the pivotal role of focused attention in the realm of object detection, particularly in complex scenarios demanding high discrimination.

### 7.3. Error Type Definitions

In the discourse of object detection, conventional metrics such as P, R, and mAP are prevalently cited. However, these metrics, while indicative of overall performance enhancements, often obfuscate the specific dimensions of improvement within a model’s architecture, rendering it challenging to pinpoint the precise areas of advancement or to assess the tangible impact of such enhancements. This limitation inherently restricts the capacity for nuanced, targeted refinements in future iterations of the model.

To transcend this constraint and furnish a more granular understanding of a model’s performance dynamics, it is imperative to dissect the components contributing to the mAP more meticulously. A stratified analysis of errors, segregating both false positives and false negatives into distinct categories, can illuminate the underlying facets of model performance. Specifically, categorizing errors into four principal types—Classification Error (accurate localization yet erroneous classification), Localization Error (correct classification marred by imprecise localization), Background Error (the misinterpretation of background elements as foreground), and Missed Error (failure to detect an object)—enables a comprehensive evaluation of the model’s enhancements.

Such a detailed error taxonomy not only clarifies the specific areas where a model excels or falters but also provides a foundational basis for targeted modifications. By elucidating the nature and distribution of errors, researchers and developers can more effectively hone their models, ensuring that subsequent enhancements address the most critical deficiencies, thereby fostering more substantive and directed advancements in object detection technology.

As delineated in Table 10, the YOLOv8-C2f-Faster-EMAv3 iteration demonstrates an elevated AP50 of 47.2, coupled with significant diminutions in classification and localization inaccuracies. Concurrently, there is a marginal uptick in background discrepancies, missed detections, and false negatives. This underscores the enhanced precision and accuracy intrinsic to the YOLOv8-C2f-Faster-EMAv3 model, while concurrently spotlighting domains such as background error mitigation that warrant further refinement. This examination furnishes pivotal insights for the meticulous enhancement of subsequent model versions, accentuating the imperative for a holistic paradigm in the evaluation and augmentation of object detection frameworks.

### 7.4. Future Work

The YOLOv8-C2f-Faster-EMAv3 model accelerates convergence and enhances perceptual capabilities through its attention mechanism and multi-scale integration, leading to better mAP performance. While it shows promising results on datasets, areas for improvement include:

1. Close Proximity Detection: The model’s effectiveness diminishes when targets of the same type are closely spaced or in sparse configurations. Enhancing the robustness and precision of the detection system is essential.
2. Generalization to Novel Shapes: The model faces challenges in generalizing to new shapes or configurations, potentially leading to decreased accuracy and more false positives. Further research is needed to overcome this limitation and boost detection efficacy.
3. Sample Balance: An imbalanced dataset may cause overfitting to specific targets. Ensuring a diverse and balanced training dataset is crucial for optimizing model performance across various scenarios.
4. Sample Quality: The efficiency of detection can be compromised by low-quality samples, such as those from low-light or occluded environments. Utilizing high-quality training data and developing strategies to counter poor sample conditions are vital for improving model resilience and accuracy in real-world conditions.

To elevate the efficacy of detection networks, it is imperative to tackle the aforementioned challenges. This encompasses ensuring a balanced distribution of data samples, refining the loss function, leveraging transfer learning methodologies, and enhancing the quality of samples through sophisticated pre-processing techniques during both training and detection phases. Pursuing advancements in these domains holds the potential to culminate in the creation of detection models of heightened robustness and precision. Such models would be adept at discerning targets across a diverse spectrum of types and size ratios, even amidst the rigors of adverse environmental conditions.

## 8. Conclusions

A novel methodology for the identification of underwater trash has been devised through the development of the YOLOv8-C2f-Faster-EMA model, crafted to surmount the hurdles of imprecise detection and diminished accuracy prevalent in aquatic environments. This innovative model enhances performance by integrating the Faster Block from FasterNet into the C2f bottleneck, thus forming the innovative C2f-Faster module. The incorporation of an EMA module and the substitution of the C2f in the backbone with the C2f-Faster-EMA module, alongside the neck section’s replacement with the C2f-Faster module, significantly amplifies the model’s efficacy.

Rigorous ablation studies and comparative analyses reveal that the YOLOv8-C2f-Faster-EMA architecture attains a mAP of 84.6%, surpassing both its predecessor and conventional object detection frameworks. These advancements not only enhance the model’s precision in recognizing underwater refuse but also contribute to a reduction in model size and an acceleration in the detection of diminutive targets.

Nonetheless, the experimental evaluation encounters limitations, notably the challenge of achieving optimal detection across all targets due to the dispersed nature of objects within the dataset. Additionally, the scarcity of datasets tailored to underwater trash curtails further refinement and performance enhancement of the model. Future endeavors aimed at augmenting the system’s proficiency will necessitate both the expansion of the dataset and continued enhancements to the model.

Subsequent experimental evaluations have corroborated the enhanced proficiency of our model in discerning diminutive objects, underscoring its adeptness and generalizability across diverse target types. Its performance extends commendably to various datasets, demonstrating a broad spectrum of applicability and potential for significant contributions within the domain of remote sensing imagery. This versatility positions our model as a promising tool for future explorations and applications in remote sensing datasets, promising to revolutionize the field with its robust detection capabilities.

## Figures and Tables

**Table 1:** Training environment and hardware platform parameters table.

Parameters	Configuration
Operational platform	Ubuntu 18.04
Compilers	Python 3.6
Network construction method	PyTorch 1.11
CPU	Intel Xeon Platinum 8350C (56 G)
GPU	NVIDIA GeForce RTX3090 (24 Gb)

**Table 2:** Some key parameters set during model training.

Parameters	Setup
Epochs	300
Batch size	16
Workers	8
Confidence	0.5
Input image size	640 × 640
Optimizer	SGD
Data enhancement strategy	Mosaic

**Table 3:** Comparison of speed of different models.

Model	GFLOPs	Latency/ms	Time/h	FPS
YOLOv8 (baseline)	8.1	0.23	3.475	109.7
YOLOv8-fasternet	10.7	0.30	3.766	98.3
YOLOv8-C2f-Faster	6.4	0.18	3.165	125.5
YOLOv8-C2f-Faster-EMA	6.6	0.19	3.434	110.6
YOLOv8-C2f-Faster-EMAv2	6.5	0.18	3.251	120.5
YOLOv8-C2f-Faster-EMAv3	6.5	0.18	3.042	129.3

**Table 4:** Improvement and promotion effect.

Model	Precision/%	Recall/%	mAP@50/%	mAP@50:95/%
YOLOv8(baseline)	72.5	75.7	79.6	53.2
YOLOv8-fasternet	73.5	78.9	81.9	52.8
YOLOv8-C2f-Faster	70.5	74.6	80.2	48.2
YOLOv8-C2f-Faster-EMA	72.4	76.9	80.8	51.4
YOLOv8-C2f-Faster-EMAv2	67.2	77.8	80.7	51
YOLOv8-C2f-Faster-EMAv3	**79.2**	**79.8**	**84.6**	**55**

**Table 5:** Comparative experiment with and without the Mosaic module.

Model	Precision/%	Recall/%	mAP@50/%
**With Mosaic**	**79.2**	**79.8**	**84.6**
Without Mosaic	72.8	76	82.4

**Table 6:** Comparison of different models.

Model	GFLOPs	Precision/%	Recall/%	mAP@50/%
Faster R-CNN	940.9	38.3	**80.4**	71.2
SSD	62.7	64.2	38.8	62.6
YOLOv7-tiny	13.2	67	77.7	79.6
YOLOv7	105.1	77.6	76.3	82.3
YOLOv8-goldyolo	10.5	84.7	73.6	83.2
YOLOv8-convnetxtv2	14.1	76.9	77.6	81.8
YOLOv8-swintransformer	79.1	61.3	75.6	77
YOLOv8-vanillanet	151.4	**90.5**	69	80.6
**Our model**	**6.5**	79.2	79.8	**84.6**

**Table 7:** Experimental results of different attention modules with YOLOv8-C2f-Faster.

Model	Precision/%	Recall/%	mAP@50/%
YOLOv8-C2f-Faster-SE	62.1	76.6	79.3
YOLOv8-C2f-Faster-CA	48.3	78.5	73.1
YOLOv8-C2f-Faster-ECA	67.6	75.9	75.3
**Our model**	**79.2**	**79.8**	**84.6**

**Table 8:** Comparison between our model and yolov8 for other target detection.

Model	Precision/%	Recall/%	mAP@50/%
YOLOv8 + Bio	1.62	1.43	1.65
Ours + Bio	**6.92**	**8.57**	**3.19**
YOLOv8 + ROV	62.8	52.5	53.8
Ours + ROV	**74.4**	**56**	**55.3**
YOLOv8 + All	49.5	44.4	45.5
Ours + All	**49.6**	**47**	**47.2**

**Table 9:** Improvement and promotion effect on the TrashCan dataset.

Model	Precision/%	Recall/%	mAP@50/%
YOLOv8(baseline)	54.5	39.3	45.8
YOLOv8-fasternet	45.8	41.3	42.9
YOLOv8-C2f-Faster	44.7	44.5	44
YOLOv8-C2f-Faster-EMA	38	43.3	41.5
YOLOv8-C2f-Faster-EMAv2	48.2	40.2	42.7
YOLOv8-C2f-Faster-EMAv3	**63.6**	**44.8**	**47.1**

**Table 10:** Improvement and promotion effects on different error types.

Model	AP_50_ ↑	E_cls_ ↓	E_loc_ ↓	E_bkg_ ↓	E_miss_ ↓	E_FP_ ↓	E_FN_ ↓
YOLOv8(baseline)	45.5	0.61	0.49	0.22	39.07	1.40	48.85
YOLOv8-C2f-Faster-EMAv3	47.2	0.49	0.25	0.36	39.93	1.43	49.41
Improvement	+1.7	−0.12	−0.24	+0.14	+0.86	+0.03	+0.56

## Data Availability

The dataset is sourced from Trash-ICRA19, and it can be downloaded from the following website. https://conservancy.umn.edu/handle/11299/214366 (10 April 2024) Another dataset is sourced from TrashCan 1.0, and it can also be downloaded from the following website. https://conservancy.umn.edu/handle/11299/214865 (10 April 2024).

## References

[1] Lebreton L.C.M., van der Zwet J., Damsteeg J.-W., Slat B., Andrady A., Reisser J. (2017). River plastic emissions to the world’s oceans. Nat. Commun..

[2] Lim X.Z. (2021). Microplastics Are Everywhere—But Are They Harmful?. Nature.

[3] Zocco F., Lin T.-C., Huang C.-I., Wang H.-C., Khyam M.O., Van M. (2023). Towards More Efficient EfficientDets and Real-Time Marine Debris Detection. IEEE Robot. Autom. Lett..

[4] Yang J., Xin L., Huang H., He Q. (2021). An Improved Algorithm for the Detection of Fastening Targets Based on Machine Vision. Comput. Model. Eng. Sci..

[5] Li C.F., Liu L., Zhao J.J., Liu X.F. (2022). LF-CNN: Deep Learning-Guided Small Sample Target Detection for Remote Sensing Classification. CMES-Comp. Model. Eng. Sci..

[6] Zou Z.X., Chen K.Y., Shi Z.W., Guo Y.H., Ye J.P. (2023). Object Detection in 20 Years: A Survey. Proc. IEEE.

[7] Girshick R., Donahue J., Darrell T., Malik J. Rich feature hierarchies for accurate object detection and semantic segmentation. Proceedings of the IEEE Conference on Computer Vision and Pattern Recognition.

[8] He K.M., Zhang X.Y., Ren S.Q., Sun J. (2015). Spatial Pyramid Pooling in Deep Convolutional Networks for Visual Recognition. IEEE Trans. Pattern Anal. Mach. Intell..

[9] Girshick R. Fast r-cnn. Proceedings of the IEEE International Conference on Computer Vision.

[10] Redmon J., Divvala S., Girshick R., Farhadi A. You only look once: Unified, real-time object detection. Proceedings of the IEEE International Conference on Computer Vision.

[11] Liu W., Anguelov D., Erhan D., Szegedy C., Reed S., Fu C.Y., Berg A.C. (2016). Ssd: Single shot multibox detector. Proceedings of the Computer Vision–ECCV 2016: 14th European Conference.

[12] Chen L., Liu Z., Tong L., Jiang Z., Wang S., Dong J., Zhou H. Underwater object detection using Invert Multi-Class Adaboost with deep learning. Proceedings of the 2020 International Joint Conference on Neural Networks (IJCNN).

[13] Jiang Z., Wang R. Underwater object detection based on improved single shot multibox detector. Proceedings of the 2020 3rd International Conference on Algorithms, Computing and Artificial Intelligence.

[14] Han F.L., Yao J.Z., Zhu H.T., Wang C.H. (2020). Marine Organism Detection and Classification from Underwater Vision Based on the Deep CNN Method. Math. Probl. Eng..

[15] Lin W.-H., Zhong J.-X., Liu S., Li T., Li G. Roimix: Proposal-fusion among multiple images for underwater object detection. Proceedings of the ICASSP 2020—2020 IEEE International Conference on Acoustics, Speech and Signal Processing (ICASSP).

[16] Liu H., Song P., Ding R. (2020). WQT and DG-YOLO: Towards domain generalization in underwater object detection. arXiv.

[17] Xu F.Q., Wang H.B., Peng J.J., Fu X.P. (2021). Scale-aware feature pyramid architecture for marine object detection. Neural Comput. Appl..

[18] Wang H., Sun S., Wu X., Li L., Zhang H., Li M., Ren P. A yolov5 baseline for underwater object detection. Proceedings of the OCEANS 2021.

[19] Wen G., Li S., Liu F., Luo X., Er M.-J., Mahmud M., Wu T. (2023). YOLOv5s-CA: A Modified YOLOv5s Network with Coordinate Attention for Underwater Target Detection. Sensors.

[20] Li J.Y., Liu C.N., Lu X.C., Wu B.L. (2022). CME-YOLOv5: An Efficient Object Detection Network for Densely Spaced Fish and Small Targets. Water.

[21] Yu H.F., Li X.B., Feng Y.K., Han S. (2023). Multiple attentional path aggregation network for marine object detection. Appl. Intell..

[22] Liu K., Peng L., Tang S.R. (2023). Underwater Object Detection Using TC-YOLO with Attention Mechanisms. Sensors.

[23] Lin T.Y., Maire M., Belongie S., Hays J., Perona P., Ramanan D., Dollár P., Zitnick C.L. (2014). Microsoft coco: Common objects in context. Proceedings of the Computer Vision–ECCV 2014: 13th European Conference.

[24] Lim J.-S., Astrid M., Yoon H.-J., Lee S.-I. Small object detection using context and attention. Proceedings of the 2021 International Conference on Artificial intelligence in information and Communication (ICAIIC).

[25] Cheng G., Yuan X., Yao X., Yan K., Zeng Q., Xie X., Han J. (2023). Towards Large-Scale Small Object Detection: Survey and Benchmarks. IEEE Trans. Pattern Anal. Mach. Intell..

[26] Li C., Guo C., Ren W., Cong R., Hou J., Kwong S., Tao D. (2019). An underwater image enhancement benchmark dataset and beyond. IEEE Trans. Image Process..

[27] Chen J., Kao S.H., He H., Zhuo W., Wen S., Lee C.H., Chan S.H.G. Run, Don’t Walk: Chasing Higher FLOPS for Faster Neural Networks. Proceedings of the IEEE/CVF Conference on Computer Vision and Pattern Recognition.

[28] Chen B.Y., Dang Z.C. (2023). Fast PCB Defect Detection Method Based on FasterNet Backbone Network and CBAM Attention Mechanism Integrated with Feature Fusion Module in Improved YOLOv7. IEEE Access.

[29] Vaswani A., Shazeer N., Parmar N., Uszkoreit J., Jones L., Gomez A.N., Kaiser L., Polosukhin I. Attention is all you need. Proceedings of the Advances in Neural Information Processing Systems.

[30] Guo M.-H., Xu T.-X., Liu J.-J., Liu Z.-N., Jiang P.-T., Mu T.-J., Zhang S.-H., Martin R.R., Cheng M.-M., Hu S.-M. (2022). Attention mechanisms in computer vision: A survey. Comput. Vis. Media.

[31] Ouyang D., He S., Zhang G., Luo M., Guo H., Zhan J., Huang Z. Efficient Multi-Scale Attention Module with Cross-Spatial Learning. Proceedings of the ICASSP 2023—2023 IEEE International Conference on Acoustics, Speech and Signal Processing (ICASSP).

[32] Davis J., Goadrich M. The relationship between Precision-Recall and ROC curves. Proceedings of the 23rd International Conference on Machine Learning.

[33] Wang G., Chen Y., An P., Hong H., Hu J., Huang T. (2023). UAV-YOLOv8: A Small-Object-Detection Model Based on Improved YOLOv8 for UAV Aerial Photography Scenarios. Sensors.

[34] Xiao X., Feng X.L. (2023). Multi-Object Pedestrian Tracking Using Improved YOLOv8 and OC-SORT. Sensors.

[35] Hu J., Shen L., Sun G. Squeeze-and-excitation networks. Proceedings of the IEEE Conference on Computer Vision and Pattern Recognition.

[36] Hou Q., Zhou D., Feng J. Coordinate attention for efficient mobile network design. Proceedings of the IEEE/CVF Conference on Computer Vision and Pattern Recognition.

[37] Wang Q., Wu B., Zhu P., Li P., Zuo W., Hu Q. ECA-Net: Efficient channel attention for deep convolutional neural networks. Proceedings of the IEEE/CVF Conference on Computer Vision and Pattern Recognition.

